# Dr. Talcott’s Address

**Published:** 1852-03

**Authors:** 


ARTICLE III.

*Dr. Talcott's Address to the Candidates for the Degree of Doctor in Medicine, in the Medical Institution of Yale College, January 15th*, 1852.

This is a plain, practical piece of advice to the gentlemen just entering upon the duties of the profession.

After urging the propriety of diligence, faithfulness, care to the welfare of the profession and morality, the author makes the following sensible remarks upon the subject of engaging in political contests:

"Let me caution you to avoid mingling in party politics. If you value your own peace and comfort, or your success in your business, never condescend to be a political partizan, or a candidate for political office. Exercise your rights as a citizen, according to your views of duty, but shun bar-room harangues, and party caucuses. Be satisfied to let the burdens of public office fall upon those shoulders that are aching to receive them, and that will probably ache still harder in sustaining them."

